# Genetic Effects on Longitudinal Changes from Healthy to Adverse Weight and Metabolic Status — The HUNT Study

**DOI:** 10.1371/journal.pone.0139632

**Published:** 2015-10-07

**Authors:** Kirsti Kvaløy, Jostein Holmen, Kristian Hveem, Turid Lingaas Holmen

**Affiliations:** HUNT Research Center, Department of Public Health and General Practice, Faculty of Medicine, Norwegian University of Science and Technology, Trondheim, Norway; Children's National Medical Center, Washington, UNITED STATES

## Abstract

**Introduction:**

The complexity of obesity and onset and susceptibility of cardio-metabolic disorders are still poorly understood and is addressed here through studies of genetic influence on weight gain and increased metabolic risk longitudinally.

**Subjects/Methods:**

Twenty seven previously identified obesity, eating disorder or metabolic risk susceptibility SNPs were tested for association with weight or metabolically related traits longitudinally in 3999 adults participating both in the HUNT2 (1995–97) and HUNT3 (2006–08) surveys. Regression analyses were performed with changes from normal weight to overweight/obesity or from metabolically healthy to adverse developments with regards to blood pressure, glucose, HDL cholesterol, triglycerides or metabolic syndrome as outcomes. Additionally, a sub-sample of 1380 adolescents was included for testing association of nine SNPs with longitudinal weight gain into young adulthood.

**Results:**

The most substantial effect on BMI-based weight gain from normal to overweight/obesity in adults was observed for the *DRD2* variant (rs6277)(OR: 0.79, 95% CI: 0.69–0.90, P = 3.9x10^-4^, adj. P = 0.015). *DRD2* was not associated with BMI on a cross-sectional level. In the adolescent sample, *FTO* (rs1121980) was associated with change to overweight at adulthood in the combined male-female sample (OR: 1.27, 95% CI: 1.09–1.49, P = 3.0x10^-3^, adj. P = 0.019) and in females (OR: 1.53, 95% CI: 1.23–1.91, P = 1.8x10^-4^, adj. P = 0.003). When testing for association to longitudinal adverse developments with regard to blood pressure, blood lipids and glucose, only rs964184 (*ZNF259/APOA5)* was significantly associated to unfavourable triglyceride changes (OR: 1.66, 95% CI: 1.36–2.03, P = 5.7x10^-7^, adj. P = 0.001). Pleiotropic effects on metabolic traits, however, were observed for several genetic loci cross-sectionally, *ZNF259/APOA5*, *LPL* and *GRB14* being the most important.

**Conclusions:**

*DRD2* exhibits effects on weight gain from normal weight to overweight/obesity in adults, while, *FTO* is associated to weight gain from adolescence to young adulthood. Unhealthy longitudinal triglyceride development is strongly affected by *ZNF259/APOA*. Our main finding, linking the *DRD2* variant directly to the longitudinal weight gain observed, has not previously been identified. It suggests a genetic pre-disposition involving the dopaminergic signalling pathways known to play a role in food reward and satiety linked mechanisms.

## Introduction

Obesity has become a major global health burden [1] with numerous comorbidities such as metabolic syndrome (MetS), type 2 diabetes and cardiovascular disease [2]. Globally, from 1980 to 2008, the prevalence of obese adults has almost doubled with an increase of 4.8% to 9.8% in men and from 7.9% to 13.8% in women, respectively [3]. In Norway, represented by the HUNT study, the prevalence of BMI-based obesity has increased from 7.7% in 1984/86 to 22.1% in 2006/08 in men and from 13.3% to 23.1% in women. Likewise, abdominal obesity has increased markedly within the same time period [4] suggesting a general increased risk of metabolic syndrome, type 2 diabetes and cardiovascular disease in this population.

Obesity and associated MetS are regarded as complex traits influenced by both environmental factors and additive genetic effects. Through twin and family studies obesity heritability estimates for obesity range between 40 and 70% has been estimated [5]. Although GWAS studies based on common genetic variants with small size effects have enabled the identification of some pathologically important genetic markers and molecular pathways [6, 7], large steps forward in elucidating the genetics of obesity are expected with more studies on rare and copy number variants with larger effect sizes [8–10]. Identification of epigenetic and environment and gene interaction (GxE) effects are further thought to contribute to unraveling the “missing heritability”[11].

The metabolic syndrome refers to a combination of traits including increased central obesity, insulin resistance, dyslipidemia and hypertension [12]. The rise in MetS prevalence is postulated to mostly be caused by changes in life style. Even so a moderate to high heritability has been found for all underlying metabolic syndrome traits and data indicate that most of the individual variation observed is due to genetic differences [13]. Genetic pleiotropy [14] has been identified with common genes reported to affect clusters of MetS traits [15, 16].

The wide-ranging genetic variants shown to be implicated in common obesity, suggest that genetic susceptibility manifested in an obesogenic environment do so through complex interactions, also implicated in systems controlling food intake such as food reward and eating behaviour [17, 18]. Several studies have shown that obese individuals behave differently with regard to food stimuli and reward compared to normal weight individuals. Previous investigations have shown that dopamine and leptin signalling pathways may be involved in the stimulation of these [19, 20]. Likewise, several recent reports discuss the common genetic grounds for obesity and drug addiction [17, 21].

Causes that influence weight gain during adulthood are incompletely understood, but thought to be affected by a complex pattern of interactions between genetic susceptibility and lifestyle. Studies have focused on candidate genes’ influence on longitudinal weight change going in both directions (ΔBMI) [22], on genetic implications on weight gain/loss in intervention studies [23] or genetic pre-disposition to weight gain during antipsychotic treatment [24]. There are indications of increased risk of unhealthy weight gain due to reduced dopamine signalling and hence weaker responsiveness to food reward [20], however, very few population based studies address the issue of genetic pre-disposition to unhealthy weight or metabolic change over time.

Investigations of factors influencing longitudinal adverse developments with regard to obesity and cardio-metabolic disorders may provide new insight into processes of timing and susceptibility important for clinical prevention strategies. Through a longitudinal design based on the HUNT Study, our main investigation included 3999 adult individuals in a study of whether genetic variants previously associated with obesity, eating behaviors and metabolic traits influence both weight gain and metabolic adverse developments longitudinally over a time period of 11 years. Our study showed that the *DRD2* variant involved in the dopaminergic signalling pathways, affects adverse weight gain in adults. The finding emphasises the importance of molecular mechanisms pre-disposing to food reward and satiety linked processes to be addressed in obesity management initiatives. Pleiotropic effects on metabolic traits were observed for several genetic loci cross-sectionally although very few genetic variants seem to influence both weight gain and adverse metabolic developments.

## Materials and Methods

### Study population and phenotypic measurements

In the HUNT Study [25] data have been collected in three different time waves, HUNT1 (1984–86), HUNT2 (1995–97) and HUNT3 (2006–08), comprising the entire adult population of the Nord-Trøndelag County in Norway. Our primary study sample included 3999 adults (47.7% men) who participated both in the HUNT2 and in the HUNT3 survey 11 years apart. Comprehensive health data questionnaires, clinical measurements and biological material were collected at both survey attendances. Another longitudinal sub-sample consisting of 1380 individuals was also included. This adolescent sample (Young-HUNT1, 45% males) had an average age of 16.0 years at baseline and 27.2 years at follow-up (HUNT3), described in detail elsewhere [26].

All examinations were done by trained nurses or technicians and weight, height and waist circumference (WC) were measured using standardised weight scales and meter bands. Height was measured to the nearest centimetre (cm), weight to the nearest 0.5 kilogram (kg) and body mass index (BMI) was calculated as weight in kg/height in m^2^. Blood pressure (BP) was measured using a Dinamap 845XT (Critikon) based on oscillometry, automatically three times per minute intervals. The average values of the two last measurements were used in our study. Total cholesterol (TC), high density lipoprotein Cholesterol (HDL-C), blood glucose (GLU) and triglycerides (TG) were measured in non-fasting serum from fresh blood samples at Levanger Hospital, Norway. Details of instruments and procedures are described previously [27, 28]. Overweight and obesity in adults were defined as having a BMI ≥ 25 or 30 kg/m^2^, respectively, or a waist circumference of ≥ 94 cm 102 cm in men and ≥ 80 cm or ≥ 88 cm in women. Overweight were assumed according to Cole et al. [29] Cases were defined as normal weight at Young-HUNT1 and overweight (BMI ≥25) at HUNT3 while controls were defined as normal weight both at Young-HUNT1 and HUNT3.

Metabolic syndrome (MetS) was defined as having at least three metabolic abnormalities for WC, blood pressure, blood glucose, HDL-cholesterol or triglycerides. Cut-offs were based on the original NCEP—ATP III definition [30]. However, as fasting measures were not available, the cut-offs for glucose and triglycerides were modified to ≥7.0 mmol/l and ≥2.1 mmol/l instead of ≥6.1 mmol/l and ≥1.7 mmol/l, respectively [2, 28]. Elevated blood pressure were defined as systolic blood pressure (SBP) ≥130 mmHg or diastolic blood pressure (DBP) ≥85 mmHg, or antihypertensive drug treatment and elevated blood glucose level as ≥7.0 mmol/l or use of or diabetes medical treatment. Decreased HDL-C levels were defined as <1.0 mmol/l in men or <1.3 mmol/l in women [31].

Pregnant women were excluded both at baseline and follow-up (n = 148 at HUNT2 and n = 6 at HUNT3) in analyses using BMI or WC. Participants treated with anti-hypertensive medication were excluded from cross-sectional and descriptive analyses where blood pressure levels were taken into account. Treatment with blood pressure lowering medication or diabetes medication, were classified as above cut-off respectively where the blood pressure and glucose measurements were used in the longitudinal association analyses.

### Ethics

All participants gave a written informed consent. The protocol was in accordance with the Helsinki Declaration approved by the Regional Committee for Ethics in Medical Research and the Norwegian Data inspectorate.

### Genotyping

DNA was extracted from peripheral blood leukocytes from EDTA whole blood or blood clots using the Gentra Purgene blood kit (QIAGEN Science, Maryland, USA). The procedure was done manually or automated with an Autopure LS (QIAGEN Science, Maryland, USA) as described by the manufacturer. To estimate associations with anthropometry (obesity/weight/height) and metabolic traits, SNPs were selected based on the most robust findings at the time of study design (year 2012) or on our own investigations [26, 32]. Genotyping was performed at CIGENE using the MassARRAY and iPlex system of the Sequenome genotyping platform (Sequenom, San Diego, CA, USA) in a SNP-multiplex design. The system uses the MALDITOF primer extension assay according to manufacturers’ recommendations. Forty ng DNA was used in the multiplex. Assays were optimised on 384 samples initially which resulted in five SNPs being excluded due to poor genotyping quality (lower call rates than 95%). This left the following 27 SNPs with their nearby gene for analyses: rs1121980 (*FTO*), rs17782313 (*MC4R*), rs11084753 (*KCTD15*), rs10838738 (*MTCH2*), rs4074134 (*BDNF*), rs569356 and rs533123 (*OPRD1*), rs35683 and rs2075356 (*GHRL*), rs6277 (*DRD2*), rs10195252 (*GRB14*), rs10242595 (*IL–6*), rs1049353 (*CNR1*), rs3782905 (*VDR*), rs3828942 (*LEP*), rs4929984 (*H19*), rs7180942 (*NTRK3*), rs8179183 (*LEPR*), rs890 (*NR2B 5073T*), rs964184 (*ZNF259/APOA5*), rs6810075 (*ADIPOQ*), rs560887 (*G6PC2*), rs12922394 (*CDH13*), rs1501299 (*Adipoq*), rs268 (*LPL*), rs782590 (*SMEK2*), rs1042725 (*HMGA2*) (S1 Table). Individuals with >10% genotype missing were removed. Two negative controls were run per 384-well plate. Samples were run blinded to the laboratory personnel.

### Statistical analyses

SNPs were tested for deviation from Hardy Weinberg Equilibrium (HWE) in the total sample (S1 Table). To test cross-sectional SNP effects, linear regression was performed on the cross-sections using BMI, WC, SBP, DBP, GLU, TC, HDL-C and TG as continuous variables. Association with WC was adjusted for height. In addition, age and sex were adjusted for in analyses of the total sample sets and age in the sex stratified analyses. Due to departure from normal distribution by right skewness, the inverse values of glucose and the lg10 values of HDL-C and TG were used. The regression analyses were performed assuming additive models for each SNP. The minor allele was used as reference.

**Fig 1:**
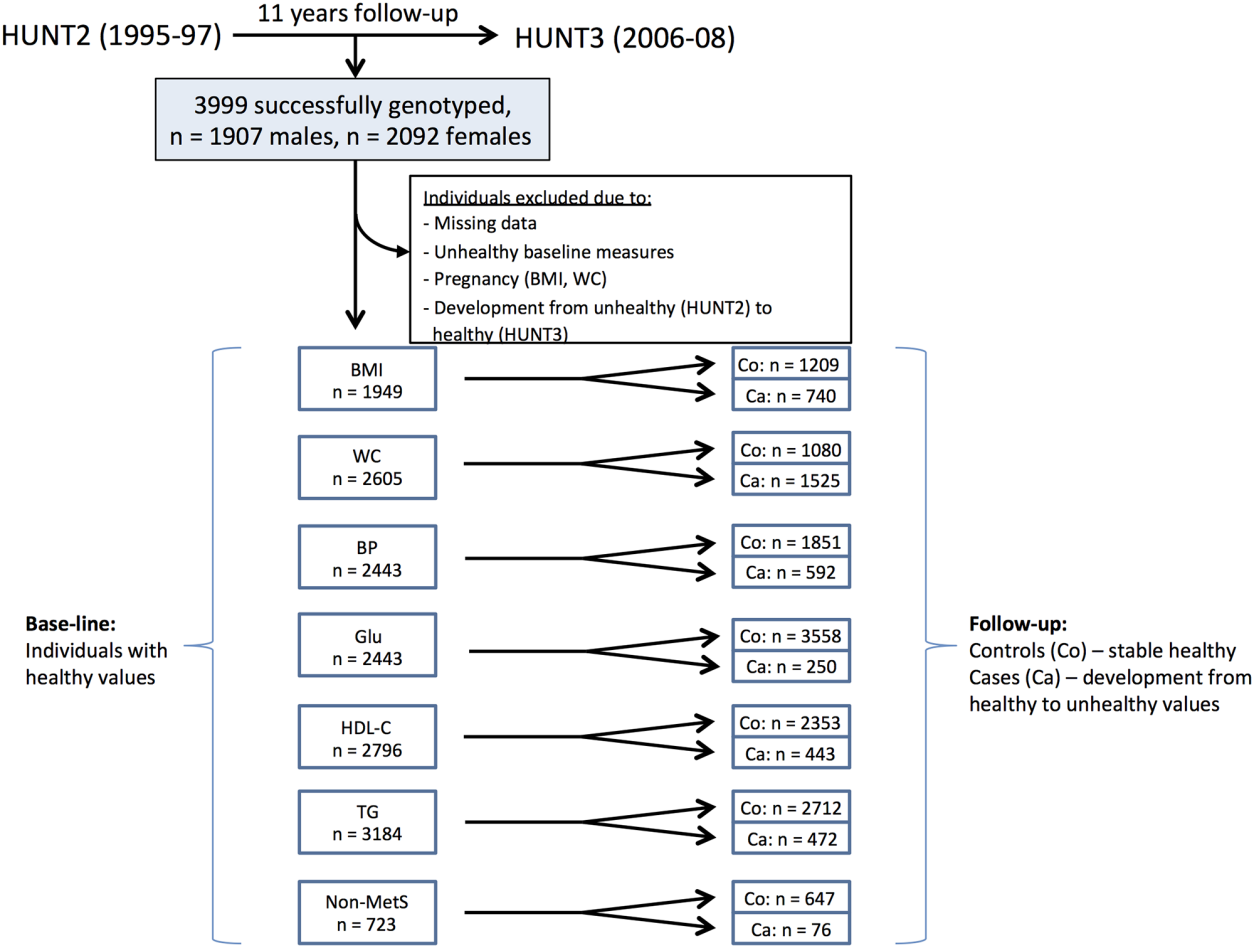
Flow diagram of individuals included in the adult longitudinal study. Overweight/obesity was defined as having a BMI ≥ 25 kg/m^2^ or as ≥ 94 cm 102 cm (male) and ≥ 80 (female) with regards to waist circumference (WC). Unhealthy blood pressure (BP) was defined as systolic blood pressure ≥130 mmHg or diastolic blood pressure ≥ 85 mmHg, or antihypertensive drug treatment. Unhealthy blood glucose (GLU) level was defined as ≥7.0 mmol/l or use of or diabetes medical treatment and triglyceride (TG) level as ≥2.1 mmol/l (both cut-offs modified due to non-fasting measurements). An unhealthy HDL cholesterol (HDL-C) level was defined below <1.0 mmol/l (male) or <1.3 mmol/l (female). Metabolic syndrome (MetS) phenotype cut-offs were based on the original NCEP—ATP III definition taking into account WC, BP, GLU, HDL-C and TG levels. MetS-cases were those scoring below cut-off for all five measures at baseline, but above cut-off for at least three components at follow-up. Controls scored below cut-offs for all five measures both at base-line and at follow-up.

Individual changes over time from HUNT2 to HUNT3 were evaluated using ANOVA repeated measures (SPSS, version 20). Additionally, association between SNPs and changes from healthy to adverse metabolic status (HUNT2 to HUNT3) in individuals with cut-offs defined above for BP/antihypertensive medication, GLU/diabetes medication, HDL-C and TG were tested by logistic regression. Likewise, logistic regression was employed testing associations between genes and longitudinal changes from normal weight to overweight (HUNT2 to HUNT3). Controls were defined as participants categorized as healthy (below cut-off) with regard to the outcome variables both at baseline and follow-up, while cases were defined as those who displayed healthy values at baseline and above cut-off metabolically or categorized as overweight at follow-up. Participants not categorized as cases or controls were excluded from the analyses (study design outlined in Fig 1). PLINK Software was used for genetic analyses [33]. Nominal significance was considered at P<0.05 and for defining sex-specific interactions (SNP*sex). A PLINK-based permutation-based test (max(T)) with 1000 permutations per analysis was used in order to adjust for multiple testing of the SNPs (equals stringency of Bonferroni correction when single SNPs are tested).

## Results

### Study subjects and phenotypic measurements

Descriptive characterisation of the adult longitudinal sample is summarised in Table 1. The sample consisted of 3999 individuals (48% males) with an average age of 35.6 years (HUNT2) at baseline and 46.8 years at follow-up (HUNT3). Comparing baseline and follow-up measures showed significant longitudinal adverse developments for most included weight and metabolic variables, however, in men HDL-C and DBP stayed nearly unchanged, while SBP significantly decreased. In contrast, DBP declined significantly over time in women. Overall overweight (BMI based) changed from 60.3% to 77.2% and 39.6% to 56.7% for men and women, respectively, and WC-based overweight from 28.9% to 62.5% and 35.6% to 77.8% for men and women, respectively. Overall obesity (BMI based) changed from 9.6% to 22.0% in men and from 10.7% to 19.4% in women. WC-based obesity changed from 7.4% to 30.5% in men and 14.7% to 51.3% in women.

**Table 1:** Descriptive characteristics of the 3999 individuals (male 48%) in the adult longitudinal study (HUNT2, 1995–97) with follow up 11 years later (HUNT3, 2006–08).

	HUNT2 (1995–97)		HUNT3 (2006–08)		P^c^	
	Male	Female	Male	Female	Male	Female
**Subject (n)**	1907	2092	1907	2092	ND	ND
**Age (years)** ^a^	37.34 (6.0)	33.98 (5.3)	48.5 (5.9)	45.16 (5.3)	ND	ND
**BMI (kg/m2)** ^a^	25.96 (3.03)	24.79 (3.93)	27.56 (3.43)	26.57 (4.66)	<0.001	<0.001
**Waist circumference** ^a^	89.82 (7.8)	77.57 (9.53)	97.04 (9.46)	88.98 (11.65)	<0.001	<0.001
**Triglycerides (mmol/L)** ^a^	1.87 (1.15)	1.21 (0.69)	1.96 (1.27)	1.28 (0.74)	<0.001	<0.001
**Total cholesterol (mmol/L)** ^a^	5.54 (1.05)	5.08 (0.99)	5.64 (1.02)	5.30 (0.98)	<0.001	<0.001
**HDL cholesterol (mmol/L)** ^a^	1.22 (0.32)	1.47 (0.35)	1.22 (0.29)	1.44 (0.33)	0.693	<0.001
**Non-fasting glucose (mmol/L)** ^a^	5.18 (0.97)	4.94 (0.76)	5.64 (1.41)	5.26 (1.20)	<0.001	<0.001
**Systolic blood pressure (mm Hg)** ^a^	130.52 (11.68)	118.47 (11.24)	129.28 (13.75)	120.11 (14.37)	<0.001	<0.001
**Diastolic blood pressure (mm Hg)** ^a^	76.14 (8.72)	70.89 (8.13)	76.46 (9.61)	69.81 (9.73)	0.152	<0.001
**Overweight, BMI ≥ 25** ^b^	1150 (60.3%)	780 (39.6%)	1470 (77.2%)	1182 (56.7%)		
**Obesity, BMI ≥ 30** ^b^	183 (9.6%)	211 (10.7%)	419 (22.0%)	404 (19.4%)		
**Overweight, WC** ^b^ ^,^ ^d^	552 (28.9%)	703 (35.6%)	1191 (62.5%)	1621 (77.8%)		
**Obesity, WC** ^b^	141 (7.4%)	290 (14.7%)	582 (30.5%)	1068 (51.3%)		

^a^Variables expressed as means ± standard deviations.

^b^Variables expressed as number of individuals and percentages.

^c^ P-value derived from pairwise comparisons (ANOVA). Inverse values for Glucose and Lg10 for HDL cholesterol and Triglyceride measurements in the ANOVA analyses. Waist circumference (WC) overweight, men ≥ 94 cm, women ≥ 80 cm. WC obesity, men ≥ 102 cm, women ≥ 88 cm.

^d^Overweight at BMI ≥ 25 includes overweight and obese individuals.

### Genetic associations with longitudinal change from normal weight to overweight

To investigate whether the genetic variants included in the study were associated with longitudinal changes from normal weight to overall overweight/obesity (BMI ≥25), a logistic regression model was employed. The sample included 740 cases (353 males, 387 females) with normal weight at HUNT2 and overweight/obesity at HUNT3 and 1209 controls (404 males, 805 females) with normal weight at both time points (Fig 1). The *DRD2* (Dopamine receptor D2)-variant (rs6277) was highly significant where the C-allele displayed a protective effect towards overweight development (OR: 0.79, 95% CI: 0.69–0.90, P = 3.9x10^-4^, adj. P = 0.015 (Table 2). Other gene variants were only significant at nominal significance levels.

**Table 2:** Association between SNPs and the longitudinal changes from normal to overweight/obesity (BMI ≥25) in the adult (HUNT2 to HUNT3) and the adolescent to adult subsamples (Young-HUNT1 to HUNT3).

					**HUNT2→HUNT3**			
					**cases: 740, controls: 120**			
**Sample**	**SNP**	**Gene**	**Ref. allele/ other allele**	**OR**	**L95**	**U95**	**P**	**P^a^**
Combined	rs560887	*G6PC2*	A/G	0.90	0.78	1.05	0.18^b^	1.00
Male				1.12	0.89	1.41	0.35	1.00
Female				0.78	0.65	0.96	0.01	0.36
Combined	rs2075356	*GHRL*	C/T	0.91	0.73	1.13	0.37^b^	1.00
Male				0.61	0.43	0.88	8.0x10 ^-3^	0.16
Female				1.13	0.86	1.49	0.37	1.00
Combined	rs268	*LPL*	G/A	1.58	1.06	2.34	0.02	0.47
Male				1.77	0.88	3.54	0.11	0.96
Female				1.49	0.92	2.42	0.11	0.95
Combined	rs4929984	*H19*	A/C	0.86	0.75	0.98	0.02	0.41
Male				0.88	0.71	1.07	0.20	1.00
Female				0.85	0.72	1.00	0.05	0.79
Combined	rs4074134	*BDNF*	A/G	0.84	0.71	0.99	0.04	0.64
Male				0.83	0.64	1.07	0.15	0.99
Female				0.86	0.69	1.06	0.16	0.99
Combined	rs10838738	*MTCH2*	G/A	1.17	1.02	1.34	0.03	0.56
Male				1.16	0.92	1.44	0.21	1.00
Female				1.20	1.00	1.43	0.05	0.79
Combined	rs6277	*DRD2*	C/T	**0.79**	**0.69**	**0.90**	**3.9x10** ^**-4**^	**0.02**
Male				0.82	0.67	1.00	0.05	0.72
Female				0.77	0.65	0.92	3.3x10^-3^	0.07
					**Young-HUNT1→HUNT3**			
					**cases: 553 controls: 827**			
Combined	rs1121980	*FTO*	T/C	**1.27**	**1.09**	**1.49**	**3.0x10** ^**-3**^ ^b^	**0.04**
Male				1.06	0.84	1.32	0.63	1.00
Female				**1.53**	**1.23**	**1.91**	**1.8x10** ^**-4**^	**<0.01**
Combined	rs17782313	*MC4R*	C/T	1.21	1.02	1.44	0.03	0.22
Male				1.16	0.91	1.49	0.22	0.89
Female				1.26	0.98	1.63	0.07	0.51

In the adult sample, cases (n = 740) were defined with normal weight at HUNT2 and overweight at HUNT3 while controls (n = 1209) were defined as normal weight both at HUNT2 and HUNT3. Overweight at adolescents were assumed according to Cole et al (2001). Cases were defined as normal weight at Young-HUNT1 and overweight (BMI ≥25) at HUNT3 while controls were defined as normal weight both at Young-HUNT1 and HUNT3. All measures were age adjusted and combined samples were additionally sex-adjusted. Empirical P-values were corrected for multiple testing by 1000 permutations.

P^a^—P-values after multiple testing. Only results with a nominal significant P-value (P<0.05, underlined) at any of the measures included are shown. Significant results after multiple testing are shown in bold.

^b^Sex interaction P<0.05.

To explore potential associations between the genetic variants and the change from normal weight to overweight/obesity (men ≥ 94 cm, women ≥ 80 cm) and normal weight to obesity (men ≥ 102 cm, women ≥ 88 cm) based on WC as outcome, logistic regression models were used. There were 1525 individuals (678 males, 847 females) in our sample that developed overweight /obesity between HUNT2 and HUNT3 and 632 obese cases (202 males, 421 females) while 1080 controls (673 males, 407 females) had WC below cut-off for overweight at both time points (Fig 1, S2 Table). The resulting associations were not generally comparable to the BMI-based results neither with regards to SNPs being identified nor the effect sizes displayed. The *DRD2* was on the border of significance with the same direction of effect in females as identified for BMI-based change to overweight. Cross-sectionally, the *FTO* (fat mass and obesity associated) variant (rs1121980) was the only marker being significantly associated in sex and age-adjusted linear regression models with BMI after multiple testing (S3 Table).

To address whether the *DRD2* showed the same longitudinal change association pattern as identified in the adults, we performed a nearly corresponding logistic regression analysis on the adolescent sub-sample. This sub-sample consisted of 553 cases (300 males and 253 females) that went from normal weight (weight categories according to Cole et al. [29]) to overweight/obese at adulthood and 827 controls (331 males and 496 females) with normal weight at both time points. Of the nine genetic variants that were included in this analysis (rs1121980 (*FTO*), rs17782313 (*MC4R*, melanocortin 4 receptor), rs11084753 (*KCTD15*, potassium channel tetramerization domain containing 15), rs10838738 (*MTCH2*, mitochondrial carrier 2), rs4074134 (*BDNF-* brain-derived neurotrophic factor), rs569356 (*OPRD1-* opioid receptor, delta 1), rs35683 (*GHRL*, ghrelin), rs6277 (*DRD2*) and rs10195252 (*GRB14*—growth factor receptor-bound protein 14)), only the *FTO* variant (rs1121980) was significant after multiple testing in the combined male-female sample (OR: 1.27, 95% CI: 1.09–1.49, P = 3.0x10^-3^, adj. P = 0.019). Additionally, sex-interaction was identified with a female-only effect observed in the stratified analyses (OR: 1.53, 95% CI: 1.23–1.91, P = 1.8x10^-4^, adj. P = 0.003) (Table 2).

### Genetic associations with longitudinal change towards adverse measures of blood pressure, blood glucose, HDL cholesterol and triglycerides

In order to examine potential genetic effects with regards to change from healthy to adverse metabolic status over time, logistic regression models were used in the same manner as for the studies of genetic effects on disadvantaged weight developments. The four different metabolic trait sub-samples included were each composed of stable healthy controls for the measure in question and cases which were healthy at HUNT2, but had developed to an adverse state at HUNT3 defined by cut-offs defined in Materials and Methods. For the blood pressure analysis 592 cases (275 males, 317 females) and 1851 (536 males, 1315 females) controls were included. The glucose based analysis involved 250 cases (164 males, 86 females) and 3558 controls (1637 males, 1921 females), HDL cholesterol 443 cases (170 males, 273 females) and 2353 controls (1107 males, 1246 females) and the triglyceride set-up 472 cases (299 males, 173 females) and 2712 controls (998 males, 1714 females)(Fig 1). Only one variant, rs964184 (*ZNF259/APOA5*, zinc finger protein 259/apolipoprotein A-V) was significantly associated with unfavourable triglyceride changes even after multiple testing (OR: 1.66, 95% CI: 1.36–2.03, P = 5.7x10^-7^, adj. P = 0.001)(S4 Table).

**Fig 2:**
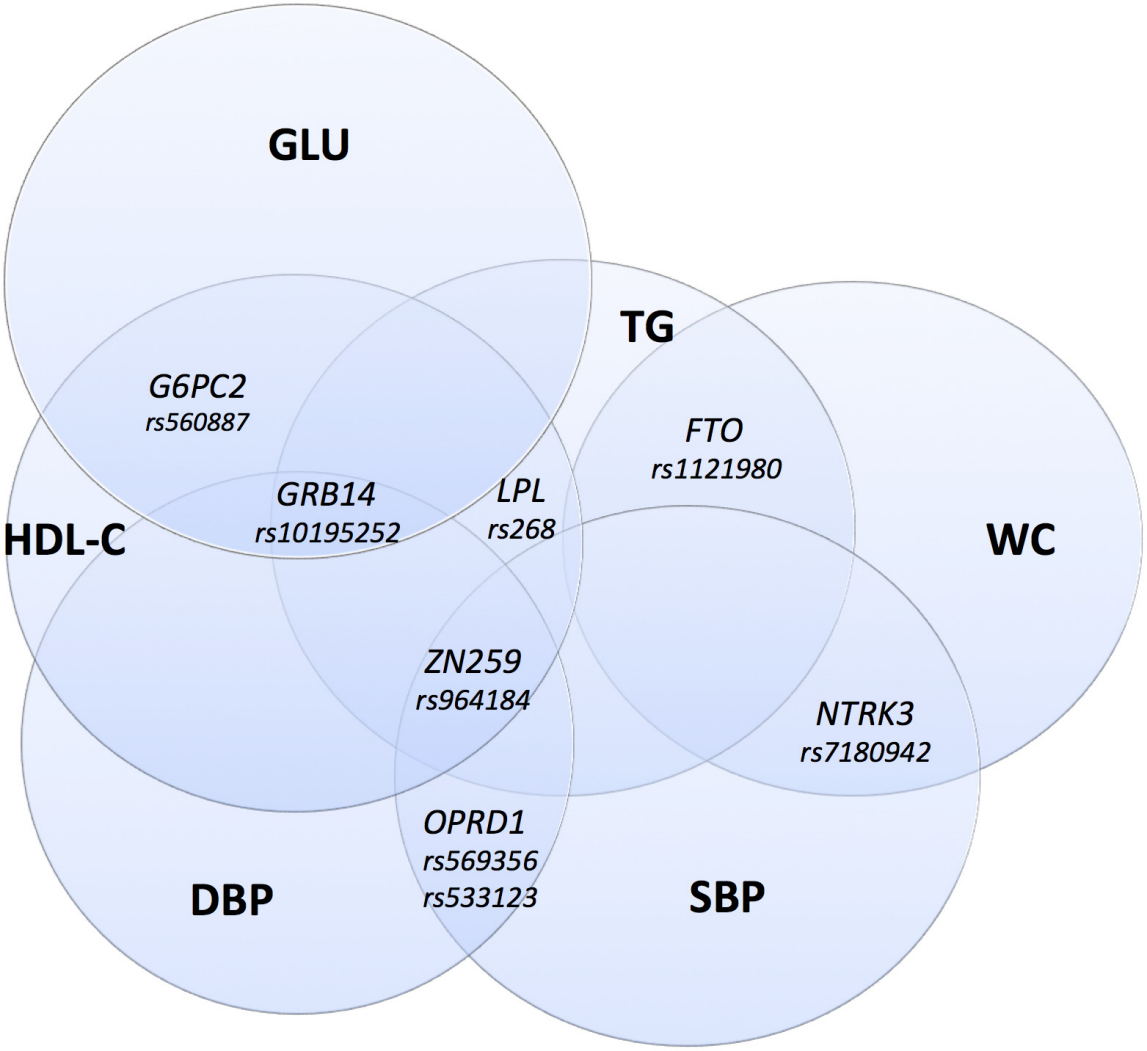
Cross-sectional associations between SNPs and metabolic syndrome (MetS) components. MetS-components: waist circumference (WC), HDL cholesterol (HDL-C), triglycerides (TG), glucose (GLU), systolic and diastolic blood pressure (SBP, DBP) at baseline (HUNT2) and follow-up (HUNT3). The following markers included were all significantly (P<0.05) associated to at least one trait cross-sectionally: *ZNF259/APOA5* (rs964184), *G6PC2* (glucose-6-phosphatase catalytic 2, rs560887), *LPL* (rs268), *GRB14* (rs10195252), *FTO* (rs1121980), *OPRD1* (rs569356 and rs533123) and *NTRK3* (neurotrophic tyrosine kinase receptor type 3, rs7180942).

Cross-sectional linear regression analyses were performed testing associations between SNPs and metabolic traits at the HUNT2 and HUNT3 time points separately. The markers *ZNF259/APOA5*, *LPL* (lipoprotein lipase) and *GRB14* displayed typical pleiotropic effects (S3 Table) further illustrated in Fig 2. The effects of rs964184 (*ZNF259/APOA5*) on TC, HDL-C and TG were significant sex-specifically with larger effects in males than in females (S5 Table).

### Genetic associations with longitudinal change from healthy to MetS positive

To explore the possibility of genetic effects influencing the longitudinal change from a healthy metabolic status to scoring positive for MetS, logistic regression was performed on 76 cases (32 males and 44 females) against 647 controls. Despite few cases, four variants displayed nominal significance. The rs560887 (*G6PC2*) was nominally significant in the combined sample and the rs533123 (*OPRD1*), rs1049353 (*CNR1*, cannabinoid receptor 1) and rs10242595 (*IL6*, interleukin 6) showed associations in males (significant sex interaction identified for *IL6* and nearly for *CNR1* (P = 0.06)). Tests for associations cross-sectionally, displayed significant results after adjustment for multiple testing with rs964184 (*ZNF259/APOA5*) at HUNT3 (OR: 1.39, 95% CI: 1.14–1.71, P = 1.6x10^-3^, adj. P = 0.031)(S6 Table).

## Discussion

In this study, genetic variants near or within 24 genes previously associated with obesity, eating behaviors and metabolic traits were used to investigate potential influence both on weight gain and metabolic adverse development over time. The study included an adult sample of 3999 and an adolescent sample of 1380 individuals whom all had participated in two HUNT surveys performed 11 years apart. The availability of prospective data and DNA enabled us to study genetic effects at two different time points as well as investigating genetic effects with potential influence on the change to overweight or an impaired metabolic status.

The *DRD2* variant was shown to influence BMI-based weight gain from normal to overweight/obesity in adults, but was not associated to BMI on a cross-sectional level. This finding was not replicated in the adolescent sub-sample. However, *FTO* showed significant association both in the combined male-female sample and in females separately. Only the *ZNF259/APOA5* variant was significantly associated to impaired metabolic changes and only with regards to triglyceride levels. Cross-sectionally, pleiotropic effects on metabolic traits were observed for several genetic variants, *ZNF259/APOA5*, *LPL* and *GRB14* being the most important.

An association between the dopamine receptor variant, *DRD2*, and overall weight change from normal to an overweight status in adults has not been reported previously. Dopaminergic signalling pathways are known to be involved in the regulation of food intake and energy expenditure through pathways involved in food reward and satiety [34] and the dopamine receptor genes (*DRD2* and *DRD4*) that impact dopamine signaling capacity have recently through functional resonance imaging (fMRI) been shown to moderate the predictive risk of unhealthy weight gain [20]. In our study, other variants previously associated with eating behavior such as *GHRL* and *BDNF* also displayed nominal significant associations to weight gain with identified sex-interaction and effect only observed in males for the *GHRL*. The observed genetic effect exerted by variants involved in neuronal pathways support the increasing amount of evidence pointing towards comparable behaviors involved in food and drug addiction as well as in reward linked mechanisms [17, 35]. As suggested by Volkow et al. [17] “genes that modulate executive control, including self-control, may help counteract the risk for overeating in food-rich environments”, which may well be the case also for the genes identified in this study. Comparable association results concerning adverse abdominal change, was in our study not significant after multiple testing. However, the same directed effect as was observed for adverse changes in BMI-based weight measures was shown for *DRD2* and another reward-related locus, *OPRD1*.

The association with reward-linked variants identified in the adult longitudinal sample was not replicated in the adolescent longitudinal sub-sample. The *FTO* obesity-risk allele was, however, shown to be associated with overweight development from adolescence to young adulthood. The different genetic effects identified in the adult and adolescent samples could be due to cohort related dissimilarities in general or that *FTO* may actually have stronger effects at a younger age. Previously, *FTO* has been shown to have its peak strength at the age of 20 before the effect weakens during adulthood [36]. The *FTO* gene is known for affecting appetite and satiety [37] and very recently this gene was reported to affect neural activity in homeostatic and brain reward regions [38] both supporting its important role in eating behavior. Interestingly, in our study the effect of *FTO* on longitudinal overweight development displayed a strong sex-interaction with the risk only being significant in females. This female-specific *FTO* effect has also been reported previously related to obesity, insulin sensitivity and glucose levels in children [39].

Recent research has shed light on how genetic variants influence combinations of components of MetS in a pleotropic fashion [16, 40]. In our study we replicated this finding for several of the genetic variants included. The G-allele of rs964184 located near the *APOA5-A4-C3-A1* gene complex, affected HDL-C and TG levels at both time points in a direction consistent with an adverse metabolic development. However, the same allele displayed protective effects on blood pressure levels with highest effects on SBP. Sex-interactions were observed for this variant when associated to TG, TC and HDL-C where the effect was male-specific in the two latter. Male specific effects of the *ZNF259/APOA5* variant on systolic blood pressure has also previously been documented [41]. Association between this variant and metabolic syndrome was identified in our study which has also been documented in a recent meta-analysis [42]. Similarly, associations between *ZNF259/APOA5* and increased levels of HDL-C and TG both in children and adults have been reported [42–45], as confirmed here at both time points in our adult sample. An interesting finding in our study was the strong sex interaction (P = 0.004) observed with the development from normal to abdominal obesity where the G-allele in the rs964184 (*ZNF259/APOA5)* revealed a risk effect in males while the opposite was the case in females. As mentioned, sex-interaction with regards to this locus has been observed before and is suggested to explain sex differences in lipid levels and their heritability [46]. The overlapping association between lipid levels and waist circumference noticed for this locus, could imply common pathophysiology between obesity and lipids traits as hypothesized previously, [46].

Lipoprotein lipase (*LPL*) is a key enzyme in lipoprotein metabolism and a major candidate gene for coronary heart disease. The previous findings of genetic association between *LPL* variants (here rs268) and blood lipids such as TG, HDL-C and TC [47–50], was confirmed in our investigation. Additionally, the interrelation that seems to exist between *ZNF259/APOA5* and *LPL* variants reported previously [47, 49], was also verified. In agreement with what was shown in the meta-analysis by Kristiansson and colleagues [42], the genetic association with MetS components seems to be lipid-driven and glucose seems to be less correlated with the other blood based MetS components.

Our main investigation comprised a population of 3999 adult individuals that could be investigated at two time points and be followed over a time period of 11 years. This quite large population sample strengthens the findings achieved on the cross-sections. However, most of the longitudinal models were underpowered due to the decision of addressing only the negatively developed outcomes. This may have precluded the identification of more significant findings. Another possible limitation of the study is the non-fasting lipid measures which may have had an effect on the case categorisation. However, recent investigations suggest that non-fasting lipid profiles change minimally in response to food intake [31]. In addition, both triglycerides and glucose cut-offs were increased according to previous investigations comparing non-fasting and fasting levels values which should preclude potentially misclassified individuals. A strength in this study was that anthropometric and clinical measurements were done by trained personnel avoiding the pitfall of under- or mis-reporting weight related measures [51] and by that underestimating the true weight gain.

The very interesting and not previously identified direct association between *DRD2* and weight gain needs to be replicated in other longitudinal studies. As large longitudinal cohorts are difficult to find we have not been able to replicate the finding at present. However, ongoing longitudinal studies such as the Norwegian Tromsø Study [52] and the Dutch study Lifelines [53] may be candidates for replications at a later stage.

In summary, we report for the first time in a population based study, a significant association between the *DRD2* gene and an overall weight gain from normal weight to overweight/obese status in adults. The same locus did not affect weight at a cross-sectional level indicating it to influence the behaviour that affects weight gain longitudinally. Interestingly, other variants also known to influence reward/addiction associated processes or eating related behaviour such as *OPRD1*, *BDNF*, *GHRL* and *CNR1*, also revealed evidence of being associated with overweight/obesity or development to an adverse metabolic status. Several metabolically related factors are influenced by common genes in a seemingly blood lipid-driven process where glucose appeared to be less correlated with the other blood based MetS components. The *ZNF259/APOA5* is an important marker in this respect, because it in addition to associate strongly to various blood lipid levels cross-sectionally at both time points investigated, also pre-dispose to a highly significant longitudinal pre-disposition to increased triglyceride levels. The identification of addiction/reward process related genes with regards to weight gain and increased metabolic risk development, state the importance of better understanding the complex neurobiology of body weight regulation in future prevention strategies.

## Supporting Information

S1 Table SNP characterisation. (DOCX)

S2 Table Association between SNPs and the longitudinal changes (HUNT2 to HUNT3) from abdominal (waist circumference, WC) normal weight to overweight/obesity or obesity. (DOCX)

S3 Table Association between SNPs and body mass index (BMI), waist circumference (WC), total cholesterol, HDL cholesterol, triglycerides, blood glucose, systolic and diastolic blood pressure at baseline (HUNT2) and follow-up (HUNT3). (DOCX)

S4 Table Associations between SNPs and the longitudinal changes from stable healthy (controls) to above cut-off in four metabolically related traits at follow-up (cases). (DOCX)

S5 Table Sex stratified associations between rs964184 (*ZNF259/APOA5*) and total cholesterol, HDL cholesterol and triglycerides at baseline (HUNT2) and follow-up (HUNT3). (DOCX)

S6 Table Association between SNPs and metabolic syndrome (MetS) both cross-sectionally and longitudinally. (DOCX)
