# Supplementary material for: Genetic Effects on Longitudinal Changes from Healthy to Adverse Weight and Metabolic Status — The HUNT Study
Source: PLoS One. 2015 Oct 7;10(10):e0139632. doi: 10.1371/journal.pone.0139632 (PMC4596824; doi:10.1371/journal.pone.0139632)
Supplement: S1 Table — (DOCX) [file pone.0139632.s001.docx]

**S1 table. SNP characterisation.**

| **CHR** | **SNP** | **Nearest gene^1^** | **Ref. allele** | **Other allele** | **MAF** | **O(HET)** | **E(HET)** | **P** |
| --- | --- | --- | --- | --- | --- | --- | --- | --- |
| 1 | rs569356 | *OPRD1* | C | T | 0.13 | 0.23 | 0.23 | 0.67 |
| 1 | rs533123 | *OPRD1* | C | T | 0.17 | 0.29 | 0.28 | 0.70 |
| 1 | rs8179183 | *LEPR* | C | G | 0.16 | 0.27 | 0.27 | 0.47 |
| 2 | rs782590 | *SMEK2* | T | C | 0.48 | 0.49 | 0.50 | 0.32 |
| 2 | rs10195252 | *GRB14* | C | T | 0.41 | 0.48 | 0.49 | 0.82 |
| 2 | rs560887 | *G6PC2* | A | G | 0.28 | 0.41 | 0.40 | 0.13 |
| 3 | rs35683 | *GHRL* | A | C | 0.43 | 0.50 | 0.49 | 0.12 |
| 3 | rs2075356 | *GHRL* | C | T | 0.10 | 0.18 | 0.18 | 0.07 |
| 3 | rs6810075 | *ADIPOQ* | C | T | 0.32 | 0.43 | 0.44 | 0.08 |
| 3 | rs1501299 | *ADIPOQ* | A | C | 0.29 | 0.41 | 0.41 | 0.44 |
| 6 | rs1049353 | *CNR1* | A | G | 0.28 | 0.41 | 0.40 | 0.48 |
| 7 | rs10242595 | *IL6* | A | G | 0.26 | 0.39 | 0.39 | 0.68 |
| 7 | rs3828942 | *LEP* | A | G | 0.43 | 0.49 | 0.49 | 0.90 |
| 8 | rs268 | *LPL* | G | A | 0.03 | 0.05 | 0.05 | 0.52 |
| 11 | rs4929984 | *H19* | A | C | 0.50 | 0.48 | 0.50 | 0.03 |
| 11 | rs4074134 | *BDNF* | A | G | 0.20 | 0.31 | 0.31 | 0.51 |
| 11 | rs10838738 | *MTCH2* | G | A | 0.35 | 0.46 | 0.46 | 0.37 |
| 11 | rs6277 | *DRD2* | C | T | 0.46 | 0.48 | 0.50 | 0.05 |
| 11 | rs964184 | *ZNF259/ APOA5* | G | C | 0.13 | 0.22 | 0.23 | 0.57 |
| 12 | rs890 | *GRIN2B* | T | G | 0.48 | 0.50 | 0.50 | 0.63 |
| 12 | rs3782905 | *VDR* | G | C | 0.32 | 0.44 | 0.44 | 0.80 |
| 12 | rs1042725 | *HMGA2* | T | C | 0.44 | 0.49 | 0.49 | 0.77 |
| 15 | rs7180942 | *NTRK3* | T | C | 0.48 | 0.51 | 0.50 | 0.09 |
| 16 | rs1121980 | *FTO* | T | C | 0.44 | 0.48 | 0.49 | 0.11 |
| 16 | rs12922394 | *CDH13* | T | C | 0.05 | 0.10 | 0.10 | 0.16 |
| 18 | rs17782313 | *MC4R* | C | T | 0.26 | 0.39 | 0.38 | 0.25 |
| 19 | rs11084753 | *KCTD15* | A | G | 0.31 | 0.44 | 0.43 | 0.22 |

CHR = chromosome location, SNP = Single Nucleotide polymorphism, Reference allele is the minor allele, MAF = Minor allele frequency, O(HET) = Observed heterozygosity, E(HET) = Expected heterozygosity, P = Hardy-Weinberg P-value. ^1^ Gene names (NCBI): *OPRD1*(opioid receptor delta 1), *LEPR* (leptin receptor), *SMEK2* (SMEK homolog 2 suppressor of mek1), *GRB14* (growth factor receptor-bound protein 14), *G6PC2* (glucose-6-phosphatase catalytic 2), *GHRL* (ghrelin), *ADIPOQ* (adiponectin C1Q and collagen domain containing), *CNR1* (cannabinoid receptor 1), *IL6* (interleukin 6), *LEP* (leptin), *LPL* (lipoprotein lipase), *H19* (H19 imprinted maternally expressed transcript), *BDNF* (brain-derived neurotrophic factor), *MTCH2* (mitochondrial carrier 2), *DRD2* (dopamine receptor D2), *ZNF259/ APOA5* (zinc finger protein 259/apolipoprotein A-V), *GRIN2B* (glutamate receptor ionotropic N-methyl D-aspartate 2B), *VDR* (vitamin D (1,25- dihydroxyvitamin D3) receptor), *HMGA2* (high mobility group AT-hook 2), *NTRK3* (neurotrophic tyrosine kinase receptor type 3), *FTO* (fat mass and obesity associated), *CDH13* (cadherin 13), *MC4R* (melanocortin 4 receptor), *KCTD15* (potassium channel tetramerization domain containing 15).
