# Supplementary material for: Genetic Effects on Longitudinal Changes from Healthy to Adverse Weight and Metabolic Status — The HUNT Study
Source: PLoS One. 2015 Oct 7;10(10):e0139632. doi: 10.1371/journal.pone.0139632 (PMC4596824; doi:10.1371/journal.pone.0139632)
Supplement: S2 Table — (DOCX) [file pone.0139632.s002.docx]

**S2 table. Association between SNPs and the longitudinal changes (HUNT2 to HUNT3) from abdominal (waist circumference, WC) normal weight to overweight/obesity or obesity.**

|  | |  | |  | **HUNT2 → HUNT3** | | | | | | | | |
| --- | --- | --- | --- | --- | --- | --- | --- | --- | --- | --- | --- | --- | --- |
|  | |  | |  | **normal → overweight/obese (WC)** | | | | | **normal → obese (WC)** | | | |
|  | |  | |  | **cases: 1525, controls: 1080** | | | | | **cases: 632, controls: 1080** | | | |
| **Sample** | **SNP** | | **Ref. allele/**  **other allele** | | | **OR** | **L95** | **U95** | **P** | **OR** | **L95** | **U95** | **P** |
| Combined | rs569356 | | C/T | | | 1.18 | 0.99 | 1.40 | 0.059 | 1.30 | 1.05 | 1.62 | 0.017 |
| Male |  | |  | | | 1.07 | 0.85 | 1.35 | 0.550 | 1.24 | 0.89 | 1.73 | 0.205 |
| Female |  | |  | | | 1.32 | 1.02 | 1.72 | 0.037 | 1.35 | 1.01 | 1.81 | 0.041 |
| Combined | rs533123 | | C/T | | | 1.08 | 0.93 | 1.25 | 0.329^b^ | 1.21 | 0.99 | 1.46 | 0.056 |
| Male |  | |  | | | 0.94 | 0.77 | 1.15 | 0.559 | 1.10 | 0.82 | 1.48 | 0.513 |
| Female |  | |  | | | 1.29 | 1.02 | 1.63 | 0.037 | 1.30 | 1.00 | 1.69 | 0.050 |
| Combined | rs6277 | | C/T | | | 0.93 | 0.84 | 1.04 | 0.224 | 0.98 | 0.85 | 1.13 | 0.762^b^ |
| Male |  | |  | | | 1.01 | 0.87 | 1.17 | 0.930 | 1.17 | 0.95 | 1.45 | 0.148 |
| Female |  | |  | | | 0.85 | 0.72 | 1.00 | 0.052 | 0.84 | 0.69 | 1.02 | 0.085 |
| Combined | rs964184 | | G/C | | | 0.99 | 0.84 | 1.17 | 0.888^b^ | 1.01 | 0.82 | 1.25 | 0.938b |
| Male |  | |  | | | 1.22 | 0.98 | 1.52 | 0.076 | 1.35 | 0.99 | 1.83 | 0.056 |
| Female |  | |  | | | 0.75 | 0.59 | 0.96 | 0.024 | 0.80 | 0.60 | 1.06 | 0.116 |

WC overweight (men ≥ 94 cm, women ≥ 80 cm). WC obesity (men ≥ 102 cm, women ≥ 88 cm). All measures were age adjusted and combined samples were additionally sex-adjusted. Only results with a nominal significant P-value (P<0.05, underlined) at any of the measures included are shown. ^b^Sex-interaction P<0.05.
