# Supplementary material for: Genetic Effects on Longitudinal Changes from Healthy to Adverse Weight and Metabolic Status — The HUNT Study
Source: PLoS One. 2015 Oct 7;10(10):e0139632. doi: 10.1371/journal.pone.0139632 (PMC4596824; doi:10.1371/journal.pone.0139632)
Supplement: S3 Table — (DOCX) [file pone.0139632.s003.docx]

**S3 table. Association between SNPs and body mass index (BMI), waist circumference (WC), total cholesterol, HDL cholesterol, triglycerides, blood glucose, systolic and diastolic blood pressure at baseline (HUNT2) and follow-up (HUNT3).**

|  | | **BMI** | | | | | | | | | **WC** | | | | | | | | | | |
| --- | --- | --- | --- | --- | --- | --- | --- | --- | --- | --- | --- | --- | --- | --- | --- | --- | --- | --- | --- | --- | --- |
|  | **HUNT2** | | | | | | **HUNT3** | | | | **HUNT2** | | | | | **HUNT3** | | | | | |
| **SNP** | | **BETA** | **95% CI** | | | **P** | **BETA** | | **95% CI** | **P** | **BETA** | **95% CI** | | **P** | | **BETA** | | **95% CI** | **P** | | |
| rs569356 | | 0.12 | -0.12, 0.36 | | | 0.32 | 0.19 | | -0.08, 0.46 | 0.17 | 0.45 | -0.12, 1.02 | | 0.12 | | 0.61 | | -0.08, 1.31 | 0.08 | | |
| rs533123 | | 0.01 | -0.20, 0.22 | | | 0.90 | 0.04 | | -0.20, 0.27 | 0.77 | 0.10 | -0.41, 0.60 | | 0.71 | | 0.25 | | -0.37, 0.86 | 0.43 | | |
| rs10195252 | | -0.01 | -0.17, 0.15 | | | 0.89 | 0.02 | | -0.16, 0.21 | 0.80 | -0.18 | -0.57, 0.20 | | 0.35 | | -0.05 | | -0.52, 0.42 | 0.84 | | |
| rs560887 | | -0.03 | -0.21, 0.15 | | | 0.76 | -0.01 | | -0.22, 0.19 | 0.90 | -0.05 | -0.48, 0.38 | | 0.81 | | -0.07 | | -0.59, 0.46 | 0.80 | | |
| rs268 | | 0.28 | -0.22, 0.77 | | | 0.27 | 0.22 | | -0.35, 0.78 | 0.46 | 0.45 | -0.74, 1.65 | | 0.46 | | 0.11 | | -1.36, 1.57 | 0.89 | | |
| rs964184 | | -0.17 | -0.41, 0.06 | | | 0.15 | -0.17 | | -0.43, 0.10 | 0.22 | -0.57 | -1.13, -0.01 | | 0.05 | | -0.59 | | -1.28, 0.10 | 0.09 | | |
| rs7180942 | | -0.16 | -0.32, -3.3x10-3 | | | 0.05 | -0.22 | | -0.40, -0.03 | 0.02 | -0.24 | -0.63, 0.14 | | 0.22 | | -0.55 | | -1.02, -0.08 | 0.02 | | |
| rs1121980 | | 0.33 | 0.17, 0.48 | | | **3.9x10^-5^** | 0.39 | | 0.21, 0.56 | **2.3x10^-5^** | 0.67 | 0.30, 1.05 | | **4.7x10^-4^** | | 0.87 | | 0.41, 1.33 | **2.0x10^-4^** | | |
|  | | **Tot cholesterol** | | | | | | | | | **HDL cholesterol** | | | | | | | | | | |
| **SNP** | | **BETA** | **95% CI** | | **P** | | **BETA** | **95% CI** | | **P** | **BETA** | **95% CI** | **P** | | | **BETA** | **95% CI** | | **P** | | |
| rs569356 | | 0.07 | 1.2x10-3, 0.13 | | 0.05 | | 0.05 | -0.02, 0.11 | | 0.16 | 2.5x10-3 | -4.3x10-3, 0.01 | 0.47 | | | 4.4x10-3 | -2.2x10-3, 0.01 | | 0.19 | | |
| rs533123 | | 0.03 | -0.03, 0.09 | | 0.28 | | 0.02 | -0.04, 0.07 | | 0.58 | 4.7x10-3 | -1.4x10-3, 0.01 | 0.13 | | | 3.9x10-3 | -2.0x10-3, 0.01 | | 0.20 | | |
| rs10195252 | | -0.09 | -0.13, -0.04 | | **1.5x10^-4^** | | -0.04 | -0.08, 0.01 | | 0.10 | 0.01 | 2.7x10-3, 0.01 | 2.0x10^-3^ | | | 0.01 | 1.3x10-3, 0.01 | | 0.01 | | |
| rs560887 | | -0.04 | -0.09, 0.01 | | 0.12 | | -0.05 | -0.10, -2.0x10-3 | | 0.04 | -2.8x10-3 | -0.01, 2.4x10-3 | 0.28 | | | -0.01 | -0.01, -2.4x10-3 | | 3.7x10^-3b^ | | |
| rs268 | | 0.06 | -0.08, 0.20 | | 0.37 | | 0.09 | -0.04, 0.23 | | 0.18 | -0.03 | -0.05, -0.02 | **5.5x10^-6^** | | | -0.03 | -0.05, -0.02 | | **8.8x10^-6^** | | |
| rs964184 | | 0.12 | 0.05, 0.18 | | **3.7x10^-4^** | | 0.15 | 0.08, 0.21 | | **6.3x10^-6^**^b^ | -0.02 | -0.02, -0.01 | **3.9x10^-7^**^b^ | | | -0.01 | -0.02, -0.01 | | **1.9x10^-5^**^b^ | | |
| rs7180942 | | -0.06 | -0.11, -0.02 | | 4.8x10^-3^ | | -0.01 | -0.06, 0.03 | | 0.60^b^ | -4.8x10-3 | -0.01, 4.2x10-3 | 0.84 | | | 6.0x10-4 | -3.9x10-3, 0.01 | | 0.79 | | |
| rs1121980 | | 0.04 | -0.01, 0.08 | | 0.11 | | 0.03 | -0.01, 0.07 | | 0.17 | -3.5x10-3 | -0.01, 1.0x10-3 | 0.13 | | | -2.0x10-3 | -0.01, 2.4x10-3 | | 0.38 | | |
|  | | **Triglycerides** | | | | | | | | | **Glucose** | | | | | | | | | | |
| **SNP** | | **BETA** | | **95% CI** | | **P** | **BETA** | **95% CI** | | **P** | **BETA** | **95% CI** | | **P** | | **BETA** | **95% CI** | | | | **P** |
| rs569356 | | 1.1x10-3 | | -0.01, 0.02 | | 0.89^b^ | -1.5x10-3 | -0.02, 0.01 | | 0.84 | -2.9x10-4 | -2.4x10-3, 1.8x10-3 | | 0.79 | | -3.5x10-4 | -2.5x10-3, 1.8x10-3 | | | | 0.75 |
| rs533123 | | -3.1x10-3 | | -0.02, 0.01 | | 0.65 | -5.0x10-4 | -0.01, 0.01 | | 0.94 | 5.0x10-4 | -1.4x10-3, 2.3x10-3 | | 0.60 | | -4.6x10-4 | -2.4x10-3, 1.5x10-3 | | | | 0.64 |
| rs10195252 | | -0.02 | | -0.03, -0.01 | | **1.3x10^-6^** | -0.02 | -0.03, -0.01 | | **9.7x10^-4^** | 1.8x10-3 | 3.8x10-4, 3.2x10-3 | | 0.01 | | -1.1x10-3 | -2.5x10-3, 3.7x10-4 | | | | 0.14 |
| rs560887 | | -3.7x10-3 | | -0.01, 0.01 | | 0.52 | -2.9x10-3 | -0.01, 0.01 | | 0.61 | 3.3x10-3 | 1.7x10-3, 4.9x10-3 | | **4.6x10^-5^** | | 2.1x10-3 | 4.4x10-3, 3.7x10-3 | | | | 0.01 |
| rs268 | | 0.06 | | 0.03, 0.09 | | **8.0x10^-5^** | 0.07 | 0.04, 0.10 | | **2.1x10^-5^** | -2.7x10-3 | 0.01, 1.8x10-3 | | 0.24b | | -1.3x10-3 | -0.01, 3.2x10-3 | | | | 0.57 |
| rs964184 | | 0.05 | | 0.04, 0.06 | | **2.2x10^-11^** | 0.06 | 0.04, 0.07 | | **2.0x10^-1^**^5b^ | 1.0x10-3 | -1.1x10-3, 3.1x10-3 | | 0.35 | | 1.3x10-3 | -8.3x10-4, 3.4x10-3 | | | | 0.23 |
| rs7180942 | | -0.01 | | -0.01, 5.0x10-3 | | 0.32 | -0.01 | -0.02, 5.8x10-4 | | 0.07 | -2.0x10-4 | -1.2x10-3, 1.6x10-3 | | 0.79 | | -2.6x10-4 | -1.7x10-3, 1.2x10-3 | | | | 0.73 |
| rs1121980 | | 0.01 | | 2.6x10-3, 0.02 | | 0.01 | 0.01 | 2.3x10-3, 0.02 | | 0.02 | -4.3x10-4 | -1.8x10-3, 9.7x10-4 | | 0.55 | | -3.5x10-4 | -1.8x10-3, 1.1x10-3 | | | | 0.63 |
|  | | **BP Systolic** | | | | | | | | | **BP Diastolic** | | | | | | | | | | |
| **SNP** | | **BETA** | | **95% CI** | | **P** | **BETA** | **95% CI** | | **P** | **BETA** | **95% CI** | | | **P** | **BETA** | | **95% CI** | | **P** | |
| rs569356 | | 1.45 | | 0.66, 2.24 | | **3.4x10^-4^** | 1.09 | 0.12, 2.06 | | 0.03 | 0.50 | -0.07, 1.07 | | | 0.09 | 0.78 | | 0.11, 1.44 | | 0.02 | |
| rs533123 | | 0.87 | | 0.1, 1.577 | | 0.02 | 1.28 | 0.42, 2.14 | | 3.5x10^-3b^ | 0.26 | -0.25, 0.76 | | | 0.32 | 0.61 | | 0.02, 1.20 | | 0.04^b^ | |
| rs10195252 | | -0.16 | | -0.70, 0.38 | | 0.56 | -0.13 | -0.78, 0.53 | | 0.70 | -0.03 | -0.42, 0.36 | | | 0.87 | 0.18 | | -0.27, 0.63 | | 0.44 | |
| rs560887 | | -0.25 | | -0.85, 0.35 | | 0.42 | 0.07 | -0.67, 0.81 | | 0.86 | -0.09 | -0.52, 0.35 | | | 0.70^b^ | 0.15 | | -0.36, 0.66 | | 0.56 | |
| rs268 | | 0.44 | | -1.2, 2.11 | | 0.61 | -0.13 | -2.17, 1.92 | | 0.90 | 0.16 | -1.05, 1.37 | | | 0.79 | 0.90 | | -0.51, 2.30 | | 0.21 | |
| rs964184 | | -1.25 | | -2.03, -0.46 | | **1.9x10^-3^** | -1.48 | -2.44, -0.52 | | **2.4x10^-3^** | -0.47 | -1.04, 0.10 | | | 0.10 | -0.84 | | -1.50, -0.18 | | 0.01 | |
| rs7180942 | | 0.27 | | -0.27, 0.80 | | 0.33 | 0.70 | 0.04, 1.35 | | 0.04 | 0.23 | -0.16, 0.61 | | | 0.25 | 0.42 | | -0.03, 0.87 | | 0.06 | |
| rs1121980 | | 0.10 | | -0.43, 0.63 | | 0.71 | 0.14 | -0.51, 0.78 | | 0.68 | -0.16 | -0.54, 0.22 | | | 0.40 | -0.18 | | -0.62, 0.27 | | 0.43 | |

All measures were age and sex- adjusted and WC additionally adjusted for height. Empirical P-values were corrected for multiple testing by 1000 permutations. Only SNPs with a near significant P-value after multiple testing at any of the two time points and for any measure are shown. Inverse values of blood glucose and the lg10 values of HDL cholesterol and triglycerides were used. P-values underlined indicate nominal significance (P<0.05). P-values in bold indicate significance after multiple testing. ^b^Sex-interaction P<0.05.
