# Supplementary material for: Genetic Effects on Longitudinal Changes from Healthy to Adverse Weight and Metabolic Status — The HUNT Study
Source: PLoS One. 2015 Oct 7;10(10):e0139632. doi: 10.1371/journal.pone.0139632 (PMC4596824; doi:10.1371/journal.pone.0139632)
Supplement: S4 Table — (DOCX) [file pone.0139632.s004.docx]

**S4 table.** **Associations between SNPs and the longitudinal changes from stable healthy (controls) to above cut-off in four metabolically related traits at follow-up (cases).**

|  | **Blood pressure** | | | | **Blood glucose** | | | | **HDL cholesterol** | | | | **Triglycerides** | | | |  |  |
| --- | --- | --- | --- | --- | --- | --- | --- | --- | --- | --- | --- | --- | --- | --- | --- | --- | --- | --- |
|  | **cases: 592, controls: 1851** | | | | **cases: 250, controls: 3558** | | | | **cases: 443, controls: 2353** | | | | **cases: 472, controls: 2712** | | | |  |  |
| **SNP** | **OR** | **L95** | **U95** | **P** | **OR** | **L95** | **U95** | **P** | **OR** | **L95** | **U95** | **P** | **OR** | **L95** | **U95** | **P** |  | |
| rs569356 | 1.23 | 1.01 | 1.50 | 0.04 | 1.11 | 0.85 | 1.45 | 0.44 | 0.96 | 0.77 | 1.19 | 0.69 | 1.00 | 0.81 | 1.24 | 0.98 |  | |
| rs35683 | 1.04 | 0.90 | 1.19 | 0.63 | 1.01 | 0.84 | 1.22 | 0.93 | 1.01 | 0.87 | 1.17 | 0.95 | 0.85 | 0.74 | 0.99 | 0.03 |  | |
| rs6810075 | 1.00 | 0.87 | 1.14 | 0.96 | 1.25 | 1.03 | 1.50 | 0.02 | 1.01 | 0.87 | 1.17 | 0.92 | 1.08 | 0.93 | 1.25 | 0.29 |  | |
| rs1501299 | 1.12 | 0.97 | 1.3 | 0.12 | 1.01 | 0.83 | 1.23 | 0.92 | 0.91 | 0.77 | 1.07 | 0.23^b^ | 0.95 | 0.81 | 1.11 | 0.49 |  | |
| rs1049353 | 0.95 | 0.82 | 1.11 | 0.51 | 1.05 | 0.85 | 1.28 | 0.66 | 0.89 | 0.76 | 1.05 | 0.16b | 0.97 | 0.83 | 1.14 | 0.74 |  | |
| rs268 | 0.56 | 0.34 | 0.92 | 0.02 | 0.75 | 0.39 | 1.44 | 0.39 | 1.49 | 0.96 | 2.31 | 0.08 | 1.64 | 1.09 | 2.47 | 0.02 |  | |
| rs10838738 | 1.05 | 0.92 | 1.21 | 0.46 | 0.94 | 0.77 | 1.14 | 0.52 | 1.17 | 1.01 | 1.36 | 0.04^b^ | 1.06 | 0.91 | 1.23 | 0.47^b^ |  | |
| rs964184 | 1.00 | 0.82 | 1.21 | 0.99 | 1.36 | 1.06 | 1.74 | 0.01 | 1.01 | 0.81 | 1.27 | 0.92 | **1.66** | **1.36** | **2.03** | **5.7x10-7^c^** |  | |
| rs7180942 | 1.01 | 0.88 | 1.15 | 0.92 | 0.96 | 0.80 | 1.17 | 0.71 | 0.94 | 0.81 | 1.09 | 0.41 | 0.86 | 0.74 | 0.99 | 0.04 |  |  |

Only SNPs with a nominal significant P-value (P<0.05) at any of the measures included are shown (underlined). Cases were defined as follows: Systolic blood pressure ≥130 mmHg or diastolic blood pressure ≥85 mmHg or antihypertensive drug treatment. Blood glucose ≥7.0 mmol/l or diabetes medication treatment. HDL cholesterol <1.0 mmol/l in men or <1.3 mmol/l in women. Triglycerides ≥2.1 mmol/l. All analyses were age and sex- adjusted. Empirical P-values were corrected for multiple testing by 1000 permutations. Results significant after multiple testing are shown in bold . ^b^Sex-interaction P<0.05. ^c^Adjusted P-value: 0.001.
