# Supplementary material for: Genetic Effects on Longitudinal Changes from Healthy to Adverse Weight and Metabolic Status — The HUNT Study
Source: PLoS One. 2015 Oct 7;10(10):e0139632. doi: 10.1371/journal.pone.0139632 (PMC4596824; doi:10.1371/journal.pone.0139632)
Supplement: S5 Table — (DOCX) [file pone.0139632.s005.docx]

**S5 table. Sex stratified associations between rs964184 (*ZNF259/APOA5*) and total cholesterol, HDL cholesterol and triglycerides at baseline (HUNT2) and follow-up (HUNT3).**

|  | |  | | **HUNT2** | | | | **HUNT3** | | | | | |
| --- | --- | --- | --- | --- | --- | --- | --- | --- | --- | --- | --- | --- | --- |
|  | | |  | **Tot Cholesterol** | | | | | | | |  |  |
| **SNP** | **Sample** | | **BETA** | | **L95** | **U95** | **P** | **BETA** | **L95** | **U95** | **P** | |  |
| rs964184 | Combined | | 0.12 | | 0.05 | 0.18 | **3.7x10^-4^** | 0.15 | 0.08 | 0.21 | **6.3x10^-6^**^b^ | |  |
|  | Male | | 0.18 | | 0.08 | 0.27 | **2.3x10^-4^** | 0.22 | 0.12 | 0.31 | **7.0x10^-6^** | |  |
|  | Female | | 0.06 | | -0.02 | 0.15 | 0.15 | 0.08 | -0.01 | 0.16 | 0.08 | |  |
|  |  | | **HDL Cholesterol** | | | | | | | | | |  |
|  |  | | BETA | | L95 | U95 | P | BETA | L95 | U95 | P | |  |
|  | Combined | | -0.02 | | -0.02 | -0.01 | **3.9x10^-7^**^b^ | -0.01 | -0.02 | -0.01 | **1.9x10^-5^**^b^ | |  |
|  | Male | | -0.03 | | -0.04 | -0.02 | **1.9x10^-8^** | -0.02 | -0.03 | -0.01 | **1.9x10^-6^** | |  |
|  | Female | | -0.01 | | -0.02 | 1.7x10-3 | 0.11 | -0.01 | -0.01 | 2.8x10-3 | 0.18 | |  |
|  |  | |  | |  |  |  |  |  |  |  | |  |
|  |  | | **Triglycerides** | | | | | | | | | |  |
|  |  | | **BETA** | | **L95** | **U95** | **P** | **BETA** | **L95** | **U95** | **P** | |  |
|  | Combined | | 0.05 | | 0.04 | 0.06 | **2.2x10^-11^** | 0.06 | 0.04 | 0.07 | **2.0x10^-15^**^b^ | |  |
|  | Male | | 0.07 | | 0.04 | 0.09 | **8.9x10^-9^** | 0.08 | 0.06 | 0.10 | **6.3x10^-14^** | |  |
|  | Female | | 0.04 | | 0.02 | 0.05 | **2.0x10^-4^** | 0.04 | 0.02 | 0.05 | **1.8x10^-4^** | |  |

Linear regression models with the combined samples were age and sex- adjusted, sex stratified models only age adjusted. Empirical P-values were corrected for multiple testing by 1000 permutations. The lg10 values of HDL cholesterol and triglycerides were used. P-values in bold indicate significance after multiple testing. ^b^Sex-interaction P<0.05.
