# Supplementary material for: Genetic Effects on Longitudinal Changes from Healthy to Adverse Weight and Metabolic Status — The HUNT Study
Source: PLoS One. 2015 Oct 7;10(10):e0139632. doi: 10.1371/journal.pone.0139632 (PMC4596824; doi:10.1371/journal.pone.0139632)
Supplement: S6 Table — (DOCX) [file pone.0139632.s006.docx]

**S6 table. Association between SNPs and metabolic syndrome (MetS) both cross-sectionally and longitudinally.**

|  | |  | | **HUNT2** | | | | **HUNT3** | | | | **HUNT2 →HUNT3** | | | |
| --- | --- | --- | --- | --- | --- | --- | --- | --- | --- | --- | --- | --- | --- | --- | --- |
|  | |  | | **cases: 373, controls: 3475** | | | | **cases: 776, controls: 3164** | | | | **cases: 76, controls: 647** | | | |
| **Sample** | **SNP** | | **OR** | | **L95** | **U95** | **P** | **OR** | **L95** | **U95** | **P** | **OR** | **L95** | **U95** | **P** |
| Combined | rs569356 | | 1.19 | | 0.95 | 1.48 | 0.13 | 1.08 | 0.91 | 1.27 | 0.37 | 1.33 | 0.80 | 2.19 | 0.27 |
| Male |  | | 1.02 | | 0.77 | 1.34 | 0.92 | 1.11 | 0.89 | 1.38 | 0.35 | 1.98 | 0.96 | 4.07 | 0.06 |
| Female |  | | 1.56 | | 1.10 | 2.23 | 0.01 | 1.04 | 0.80 | 1.34 | 0.79 | 0.99 | 0.47 | 2.06 | 0.97 |
| Combined | rs533123 | | 1.01 | | 0.83 | 1.24 | 0.90 | 1.07 | 0.92 | 1.24 | 0.36 | 1.31 | 0.85 | 2.01 | 0.22 |
| Male |  | | 0.91 | | 0.71 | 1.17 | 0.47 | 1.06 | 0.87 | 1.29 | 0.55 | 1.92 | 1.02 | 3.62 | 0.04 |
| Female |  | | 1.24 | | 0.88 | 1.74 | 0.23 | 1.08 | 0.86 | 1.36 | 0.50 | 1.00 | 0.55 | 1.83 | 1.00 |
| Combined | rs8179183 | | 0.75 | | 0.60 | 0.95 | 0.02 | 0.94 | 0.80 | 1.10 | 0.43 | 0.83 | 0.51 | 1.35 | 0.46 |
| Male |  | | 0.79 | | 0.60 | 1.03 | 0.08 | 0.85 | 0.69 | 1.05 | 0.12 | 0.67 | 0.29 | 1.52 | 0.34 |
| Female |  | | 0.68 | | 0.45 | 1.05 | 0.08 | 1.07 | 0.84 | 1.36 | 0.58 | 0.93 | 0.51 | 1.71 | 0.82 |
| Combined | rs10195252 | | 0.88 | | 0.75 | 1.03 | 0.12 | 0.88 | 0.79 | 0.99 | 0.04 | 1.03 | 0.73 | 1.44 | 0.87 |
| Male |  | | 0.88 | | 0.72 | 1.06 | 0.18 | 0.84 | 0.72 | 0.98 | 0.03 | 1.23 | 0.72 | 2.12 | 0.45 |
| Female |  | | 0.88 | | 0.67 | 1.16 | 0.38 | 0.93 | 0.78 | 1.11 | 0.42 | 0.93 | 0.60 | 1.44 | 0.74 |
| Combined | rs560887 | | 0.93 | | 0.78 | 1.11 | 0.45^b^ | 1.12 | 0.99 | 1.27 | 0.07 | 1.52 | 1.07 | 2.17 | 0.02 |
| Male |  | | 1.09 | | 0.89 | 1.34 | 0.40 | 1.12 | 0.95 | 1.32 | 0.17 | 1.48 | 0.86 | 2.55 | 0.15 |
| Female |  | | 0.65 | | 0.46 | 0.91 | 0.01 | 1.14 | 0.94 | 1.39 | 0.19 | 1.49 | 0.93 | 2.39 | 0.10 |
| Combined | rs6810075 | | 0.90 | | 0.77 | 1.06 | 0.22^b^ | 1.10 | 0.98 | 1.24 | 0.10 | 1.09 | 0.77 | 1.53 | 0.64 |
| Male |  | | 0.75 | | 0.61 | 0.93 | 0.01 | 1.08 | 0.92 | 1.26 | 0.34 | 0.90 | 0.52 | 1.56 | 0.70 |
| Female |  | | 1.24 | | 0.95 | 1.63 | 0.11 | 1.13 | 0.95 | 1.35 | 0.18 | 1.22 | 0.78 | 1.89 | 0.38 |
| Combined | rs1049353 | | 0.91 | | 0.76 | 1.08 | 0.29^b^ | 0.99 | 0.88 | 1.13 | 0.92 | 0.74 | 0.49 | 1.11 | 0.15 |
| Male |  | | 1.02 | | 0.83 | 1.26 | 0.84 | 0.98 | 0.83 | 1.16 | 0.83 | 0.44 | 0.21 | 0.92 | 0.03 |
| Female |  | | 0.71 | | 0.52 | 0.98 | 0.04 | 1.02 | 0.84 | 1.24 | 0.85 | 0.98 | 0.61 | 1.59 | 0.95 |
| Combined | rs10242595 | | 0.99 | | 0.83 | 1.18 | 0.90 | 0.93 | 0.82 | 1.06 | 0.30 | 0.88 | 0.59 | 1.32 | 0.54^b^ |
| Male |  | | 1.00 | | 0.81 | 1.24 | 0.97 | 0.89 | 0.75 | 1.06 | 0.18 | 0.44 | 0.22 | 0.90 | 0.03 |
| Female |  | | 0.96 | | 0.71 | 1.31 | 0.82 | 1.00 | 0.82 | 1.22 | 1.00 | 1.28 | 0.77 | 2.11 | 0.34 |
| Combined | rs4929984 | | 1.16 | | 1.00 | 1.35 | 0.06 | 0.99 | 0.88 | 1.10 | 0.79 | 1.00 | 0.73 | 1.40 | 0.95 |
| Male |  | | 1.22 | | 1.02 | 1.47 | 0.03 | 1.09 | 0.94 | 1.26 | 0.26 | 1.10 | 0.73 | 2.05 | 0.45 |
| Female |  | | 1.03 | | 0.79 | 1.34 | 0.82 | 0.86 | 0.72 | 1.02 | 0.08 | 0.87 | 0.57 | 1.33 | 0.51 |
| Combined | rs964184 | | 1.29 | | 1.04 | 1.59 | 0.02 | 1.24 | 1.05 | 1.45 | 0.01 | 1.05 | 0.63 | 1.76 | 0.85 |
| Male |  | | 1.46 | | 1.14 | 1.88 | 2.7x10^-3^ | **1.39** | **1.14** | **1.71** | **1.6x10^-3^** | 1.49 | 0.67 | 3.30 | 0.32 |
| Female |  | | 0.93 | | 0.62 | 1.41 | 0.74 | 1.02 | 0.79 | 1.33 | 0.86 | 0.85 | 0.43 | 1.71 | 0.66 |
| Combined | rs17782313 | | 1.19 | | 1.01 | 1.42 | 0.04 | 1.07 | 0.94 | 1.22 | 0.28 | 1.04 | 0.69 | 1.55 | 0.86 |
| Male |  | | 1.25 | | 1.02 | 1.54 | 0.03 | 1.14 | 0.97 | 1.35 | 0.12 | 0.75 | 0.37 | 1.54 | 0.44 |
| Female |  | | 1.10 | | 0.81 | 1.49 | 0.56 | 1.00 | 0.82 | 1.22 | 0.98 | 1.20 | 0.74 | 1.95 | 0.47 |

Only SNPs with at least a nominal significant P-value (P<0.05) at any level are included. MetS cases were defined as scoring above cut-off on at least three of the following five traits: 1) Waist circumference: men ≥ 102 cm, women ≥ 88 cm. 2) Systolic blood pressure ≥130 mmHg or diastolic blood pressure ≥85 mmHg or antihypertensive drug treatment. 3) Blood glucose ≥7.0 mmol/l or diabetes medical treatment. 4) HDL cholesterol <1.0 mmol/l in men or <1.3 mmol/l in women. 5) Triglycerides ≥2.1 mmol/l. Analyses were age and sex- adjusted and sex stratified analyses only age adjusted. Empirical P-values were corrected for multiple testing by 1000 permutations. P-values underlined indicate nominal significance, P-values in bold significance after multiple testing. ^b^Sex-interaction P≤0.05.
